# A Watermarking Scheme for High Efficiency Video Coding (HEVC)

**DOI:** 10.1371/journal.pone.0105613

**Published:** 2014-08-21

**Authors:** Salahuddin Swati, Khizar Hayat, Zafar Shahid

**Affiliations:** 1 COMSATS Institute of Information Technology, Abbottabad, Pakistan; 2 College of Arts and Sciences, University of Nizwa, Nizwa, Oman; University of Catania, Italy

## Abstract

This paper presents a high payload watermarking scheme for High Efficiency Video Coding (HEVC). HEVC is an emerging video compression standard that provides better compression performance as compared to its predecessor, i.e. H.264/AVC. Considering that HEVC may will be used in a variety of applications in the future, the proposed algorithm has a high potential of utilization in applications involving broadcast and hiding of metadata. The watermark is embedded into the Quantized Transform Coefficients (QTCs) during the encoding process. Later, during the decoding process, the embedded message can be detected and extracted completely. The experimental results show that the proposed algorithm does not significantly affect the video quality, nor does it escalate the bitrate.

## Introduction

High Efficiency Video Coding (HEVC) is a relatively new video compression standard, developed by the Joint Collaborative Team on Video Coding (JCT-VC), from ITU-T VCEG and ISO/IEC MPEG [1]. The main goals of HEVC design include increased video resolutions and the exploitation of parallel processing architectures [2]. HEVC is suited for a variety of applications, such as broadcast of high definition (HD) TV signals over satellite, terrestrial transmission systems and cables, video content acquisition and editing systems, security applications, camcorders, Blue-ray discs, Internet and mobile network video and real-time conversational applications that include video conferencing, video chat, and tele-presence systems [3]. One of the downside, of the ever-growing nature of Internet and multimedia technologies, is the high risk associated with the ease of manipulation, tampering and illegal copying of the digital contents, especially the multimedia. The security of digital contents, therefore, constitutes a quintessential aspect of copyright protection in today's multimedia related industries. For this very reason, the integrity, verification and authentication of digital videos form an active research area today [4]. Of special interest is the field of digital watermarking wherein the owner's/consumer's watermark is digitally embedded in the digital content, for protection against unauthorized copying as well as the ownership declaration and contents authorization [5], [6].

Digital watermarking of HEVC encoded videos may be a difficult task, because the codec eliminates most of the redundancy that the watermarking process may exploit. Casual embedding of a watermark, thus, may escalate the final video file size or otherwise affect the quality of the video; a carefully conceived embedding strategy is thus needed. Keeping these in view, we intend to propose an HEVC watermarking scheme that would have negligible effect on both the video quality and the final file size. Our strategy is to embed a watermark, during the encoding process, that can be completely extractable during the decoding process. Normally, a watermark may either be embedded in the spatial domain or the frequency domain. With spatial domain video watermarking, the hidden data may be lost during the quantization step of the underlying video codec. One solution to this problem is to embed the watermark in such a way that it survives the quantization loss. But this may come at the cost of lower imperceptibility. A better solution is to go for the frequency domain and better embed the watermark after the quantization step, i.e. in the quantized transform coefficients (QTCs). In our approach, we adopt this later approach and embed the watermark message in the selected non-zero QTCs of all the frames of the video.

The rest of the paper is organized as follows. For a better comprehension of this article, the first part of Section outlines a brief overview of the state of the art HEVC standard. The second part of the same section provides a brief literature review regarding the watermarking techniques proposed for various video coding standards, in vogue. The proposed watermarking algorithm is outlined in Section 0.2.2, wherein both the embedding and detection processes are described. Section 0.2.2 analyses the experimental results, followed by the concluding remarks in Section 0.2.2.

## Previous Work

### 0.1 An Overview of HEVC

**Figure 1:**
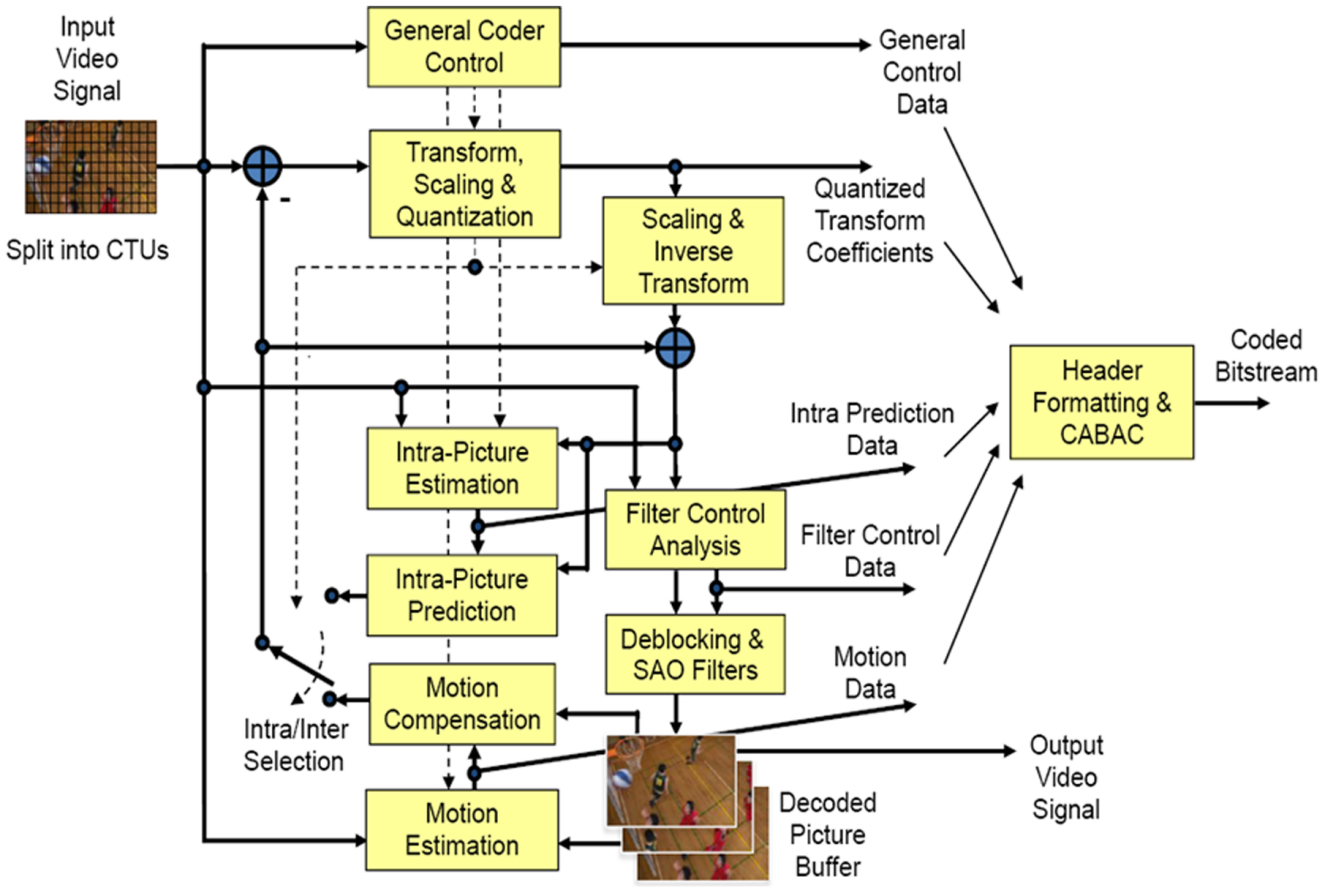
Block Diagram of HEVC [2].

Like its recent predecessors, HEVC is also a hybrid video compression standard based on the Intra/Inter Prediction and a 2D transform. It is an effort to improve upon the existing tools used in H.264. Besides, many new coding tools have been introduced in the HEVC; the most important change being its frame partitioning. Figure 1 illustrates a block diagram of the HEVC encoding process. Following are the salient features of HEVC:

1. HEVC introduces the three new concepts of Coding Unit (CU), Prediction Unit (PU) and Transform Unit (TU).
2. The coding pipeline splits each frame into what are called Coding Tree Units (CTU). A CTU has one Coding Tree Block (CTB) covering a 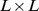 luma block and the corresponding 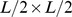 chroma blocks. The size of luma, L, may refer to 64, 32 or 16 samples.
3. A CTB can be partitioned into smaller blocks using a Quad Tree structure. A given CU is a part of CTB and can be divided recursively into 4 CUs and each has an associated division into Prediction Unit (PU) and Transform Unit(TU).
4. PU is created when a prediction method is chosen. The information of the prediction method (Intra/Inter and the related data) is contained in the PU.
5. The Prediction Block can be split using a sampling scheme that may range 64×64 to 4×4 samples.
6. For the intra-prediction in HEVC, 33 angular directions are used.
7. The PU can be sub divided into 2 rectangular or 4 square partitions, in the inter-prediction. For motion compensation, the PU division may be unidirectional or bi-directional.
8. HEVC uses transform coding of the prediction residual in the similar way as its predecessor H.264/AVC. The residual block is split into smaller square transform blocks (TBs).
9. The transform is an approximation of DCT its block sizes can be 32×32, 16×16, 8×8 and 4×4.
10. HEVC also has mode dependent alternative transform. An alternative integer transform derived from discrete sine transform (DST) is applied on each TB of size 4×4. The DST is only applied on luma transform blocks.
11. Transform coefficients in the encoder side are quantized to limit the number of bits. At the start, the quantization level is defined by a quantization parameter (QP) value that controls the uniform-reconstruction quantization (URQ) scheme. To further decrease the bitrate, the QTCs are entropy coded.
12. Only one entropy coding is specified in the HEVC, i.e. the context adaptive binary arithmetic coding (CABAC). The CABAC is used to encode the first coefficients (levels), Golomb-Rice coding to code the next and Exponential-Golomb coding is employed for encoding the last levels.
13. The degradation of frames, which is caused by compression, is restored by applying three kinds of filters namely the sample adaptive offset (SAO), de-blocking and adaptive loop filters (ALF).
14. A new video parameter set (VPS) is also introduced in HEVC.
15. To increase the parallel processing capability, HEVC introduces three new features other than the slices, such as tiles, wavefront parallel processing (WPP) and dependent slices.

It is pertinent to note that H.264 concepts, like the high level syntax and the Network Abstraction layer (NAL), are being retained in HEVC.

### 0.2 Related literature

While being still in its rudimentary phase, efforts regarding the watermarking of HEVC videos are scarce. The literature is, however, replete with algorithms regarding the watermarking of videos based on H.264 and other coding standards. With H.264, the watermarks are normally embedded into the DCT coefficient from the I- and P-frames [7]–[13]. Still, there are many methods that rely on the motion vectors (MVs) - rather than the DCT coefficients - for embedding in the compressed video domains and are usually classified as MV-based watermarking schemes [10], [14]–[19]. The embedded watermark may either be detected/extracted from partially decoded video [7], [10]–[12] or from completely decoded video [8], [9].

#### 0.2.1 DCT based methods

Zhang et al. [7] propose a robust scheme for H.264/AVC based on the spread spectrum watermarking. In this scheme a 2D-8 bit watermark message (logo) is converted into a binary sequence, and then the watermark message is embedded into the middle frequencies, i.e. the diagonal portion of the corresponding 4X4 DCT block. In another robust method, by Noorkami et al. [8], the watermark is embedded in the QTCs of I-frames. This method requires entropy decoding for embedding the watermark. For handling the visual degradation, the method looks into the human visual model. While using a key dependent algorithm, the message is embedded in a selected subset of the coefficients with reasonable visual watermarking capacity. In [9], the watermarking involves the nonzero quantized AC residuals in the P-frames. The authors have shown that the visual quality of video is not compromised even if all the non-zero quantized AC residuals are used to embed the watermark. This scheme may, however, affect the performance of the context adoptive variable length coding (CAVLC), which may in turn increase the bit-rate, due to the presence of many non-zero quantized AC residuals with the value of 1; CAVLC encodes the trailing ones (T1s) separately. In a related method [20] the watermark is embedded in the sign bit of the T1s in CAVLC. The advantage of this technique is that it does not increase the bitrate. The main disadvantage of these schemes is that their payload is very low. Besides, these are not robust to re-encoding with different parameters. The blind scheme of [12] embeds the watermark into the syntactic elements of H.264 compressed bitstream in order to avoid full decoding during both the embedding and extraction. The scheme exploits the 4×4 intra prediction submacroblocks of Luma components from the I-frames. The H.264/AVC fingerprinting technique, in [13], employs the Tardos fingerprinting codes [21] for the underlying spread spectrum robust embedding technique. In [22], a combined watermarking and encryption scheme is presented for H.264/AVC and HEVC. In this scheme, an end to end commutative security system for video distribution is proposed. The authors have investigated the trade off between robust watermarking, encryption scheme security and transcoding possibilities. The watermark is embedded into the DCT coefficient using the quantization index modulation system.

The MPEG-2/4 based Watermarking methods, from the literature, also rely on the DCT coefficients [23]–[26]. One such method [23] embeds the watermark into the DCT coefficients of the compressed video stream, whereas the watermark detection is performed using the uncompressed video. In one blind scheme [25], the watermark message is embedded in the bit-stream of MPEG-2 without affecting the bit-rate. In [24], the message is embedded into the video by pseudorandomly selecting the macroblocks (MBs) from every luminance block. It selects MBs and QTC pairs, to be modified, and then computes a frequency mask for each selected MB. This is followed by the use of this mask to weigh the watermark amplitude and then modify the selected middle frequency QTCs to carry the watermark information. The differential energy watermarking (DEW) algorithm [26] is based on the selective discard of high frequency DCT coefficients in the compressed data stream. This real-time method encodes the label bits in the pattern of energy difference between the DCT blocks. The message is embedded bit by bit in a set of an 8×8 DCT blocks from the I-frames of the MPEG compressed video stream.

#### 0.2.2 Motion vector based methods

In MV based watermarking schemes, the watermark is embedded either directly in the video bitstream [14]–[16], [19] or during the video encoding process [10], [17], [18]. The watermark is usually extracted from partly decoded video. A method, for H.264 video streams [14], hides the copyright information in proper motion vector (MV) component that considers the movement direction in the underlying video. An adaptive threshold, used to select the required MVs, determines the number of bits to be embedded. In [15], the message bits are embedded in the two least significant bit (LSBs) of the larger component from the MVs of H.264 video. The payload of this scheme is very low, however. The technique of [16], for MPEG, hides the copyright information in larger magnitude MVs, especially those with low phase angle change. The scheme is fragile having limited payload. In [17], first the luminance component of P frame is divided into low-texture and high-texture area and then MVs are modified according to the texture of the area. The prediction errors of the matched blocks are calculated again according to the changed MVs. Finally, the new MVs along with new predicted errors are encoded. In one Audio Video Coding Standard (AVS) oriented method, the message embedding is performed by altering the resolution of MVs, from the partition blocks in different MB partitions, during the inter-prediction stage of AVS. The modulation is based on the mapping rules between MV resolution and message bits. The water scrambling scheme of [19] is based on the MPEG compression scheme wherein the MVs are extracted in two ways. In the first, the MVs are extracted from MPEG bitstream using a syntactic analyzer while, in the second, the MVs are directly modified during the MPEG compression.

Beside the DCT based and MV-based strategies, there are approaches, like [27], which embed watermark in H.264 by using the intra-prediction. It is a stream replacement scheme for video watermarking. and changes the H.264 encoded bitstream for watermarking. All such schemes notwithstanding, references regarding the HEVC watermarking are almost non-existent in the literature, mainly due to its early stages of development.

## The Proposed Watermarking Scheme for HEVC

**Figure 2:**
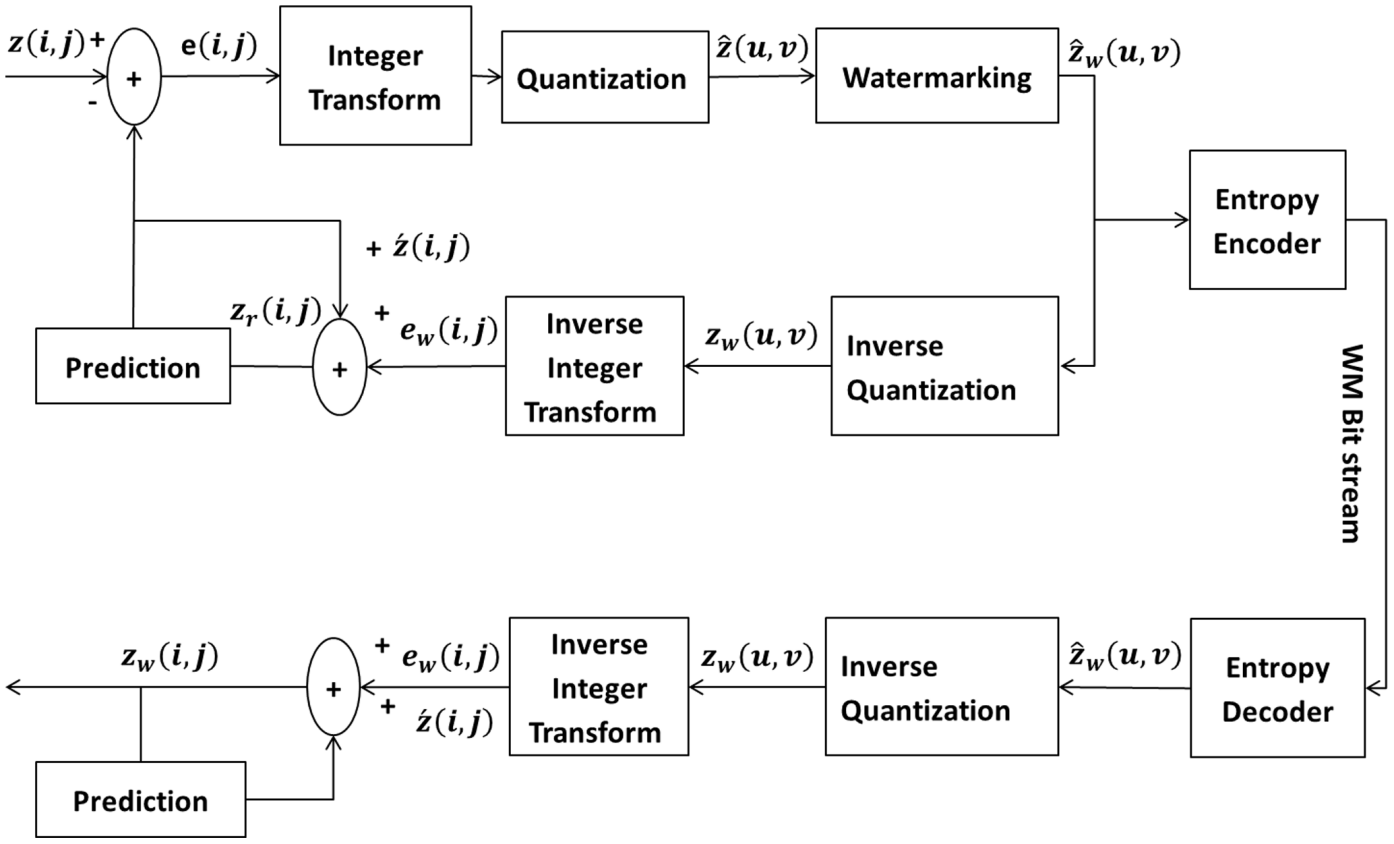
The Proposed Watermarking scheme for HEVC.

The proposed algorithm targets mainly the imperceptibility of the cover and it can be employed in applications where robustness is of secondary importance, e.g. broadcasting and hiding some sort of metadata. For embedding, we are relying on the LSB modification of the QTCs from the HEVC coding pipeline. The watermark is embedded in the coefficients whose values agree to a certain predefined threshold. The value of the threshold is selected on the basis of the size of the watermark, in bits. Figure 2 outlines the proposed watermarking scheme for HEVC. We consider the following points while embedding the message in LSB of QTCs:

- To avoid any significant escalation in the compression efficiency, only the non-zero QTCs are being considered for embedding - otherwise, many zero magnitude coefficient may become non-zero in the embedding process, thereby affecting the zero runlengths.
- The message is embedded should be completely extractable on the decoder side.

The proposed algorithm modifies the LSB of the selected QTCs and embeds one of the watermark bit (*M_b_*) in each QTC. The selection criteria for QTC is based on a threshold value of 1; if the absolute value of QTC is superior to this threshold then a watermark bit is embedded in its LSB, as demonstrated by the algorithm illustrated in Table 1. The watermark embedding function 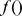 has thus two inputs, 1) a subset of QTCs (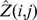) and 2) the watermark message (*M*) composed of bits *M_b_*. The watermarked QTCs are denoted by 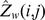 and are given by Eq. 4.
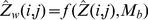
(1)


**Table 1:** Watermark embedding Algorithm.

	Input: QTC 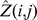 and Watermark bit *M_b_*
	Output: Watermarked QTC, 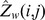
1.	**begin**
2.	**if** 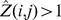
3.	**then**
4.	**set** 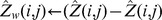 mod 2)+*M_b_*
5.	**replace** 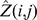 by 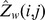
6.	**end if**
7.	**end**

The decoding function, 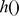, is blind and needs only the watermarked QTCs - 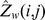 - in order to extract the watermark bits *M_b_* as shown in Eq. 2.
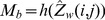
(2)


The extraction of watermarked bit is illustrated by the algorithm of Table 2.

**Table 2:** Watermark Extraction Algorithm.

	Input: Watermarked QTC, 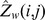
	Output: Watermark bit *M_b_*
1.	**begin**
2.	**if** 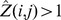
3.	**then**
4.	**set** 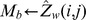 mod 2
5.	**end if**
6.	**end**

## Experimental Results

The proposed watermarking algorithm had been applied to benchmark video sequences of various resolutions. These video sequences are listed in Table 3, along with their resolutions and frames per second (FPS). The evaluation was based on a sample of 100 frames from each video and involved QP values of 18 and 32 [28].

**Table 3:** Sample video sequences used to evaluate the performance of proposed watermarking scheme.

Videos	Resolution	FPS
PeopleOnStreet	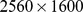	30
ParkScene	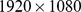	30
Chinaspeed	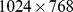	30
Vidyo1	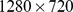	60
BQMall	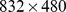	30–60
RaceHorses	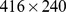	30–60

The presence non-zero coefficients, corresponding to a given frame, is usually attributed to the texture and edges. Being spatial masking parts in the frame, these areas are good candidates for the watermark embedding as far as the conservation of the compression ratio is concerned. The downside, however, may be the ensued negative impact on imperceptibility; In our case, this effect is minimized due to LSB embedding. Peak Signal to Noise Ratio (PSNR) measure has been used to analyze the quality of watermarked video with respect to original video which is given by:
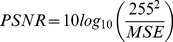
(3)


Where mean square error (MSE) is a measure used to quantify the difference between the initial video frame I and the distorted video frame 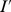. If the video frame has a size of M x N then:
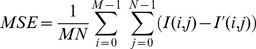
(4)


**Figure 3:**
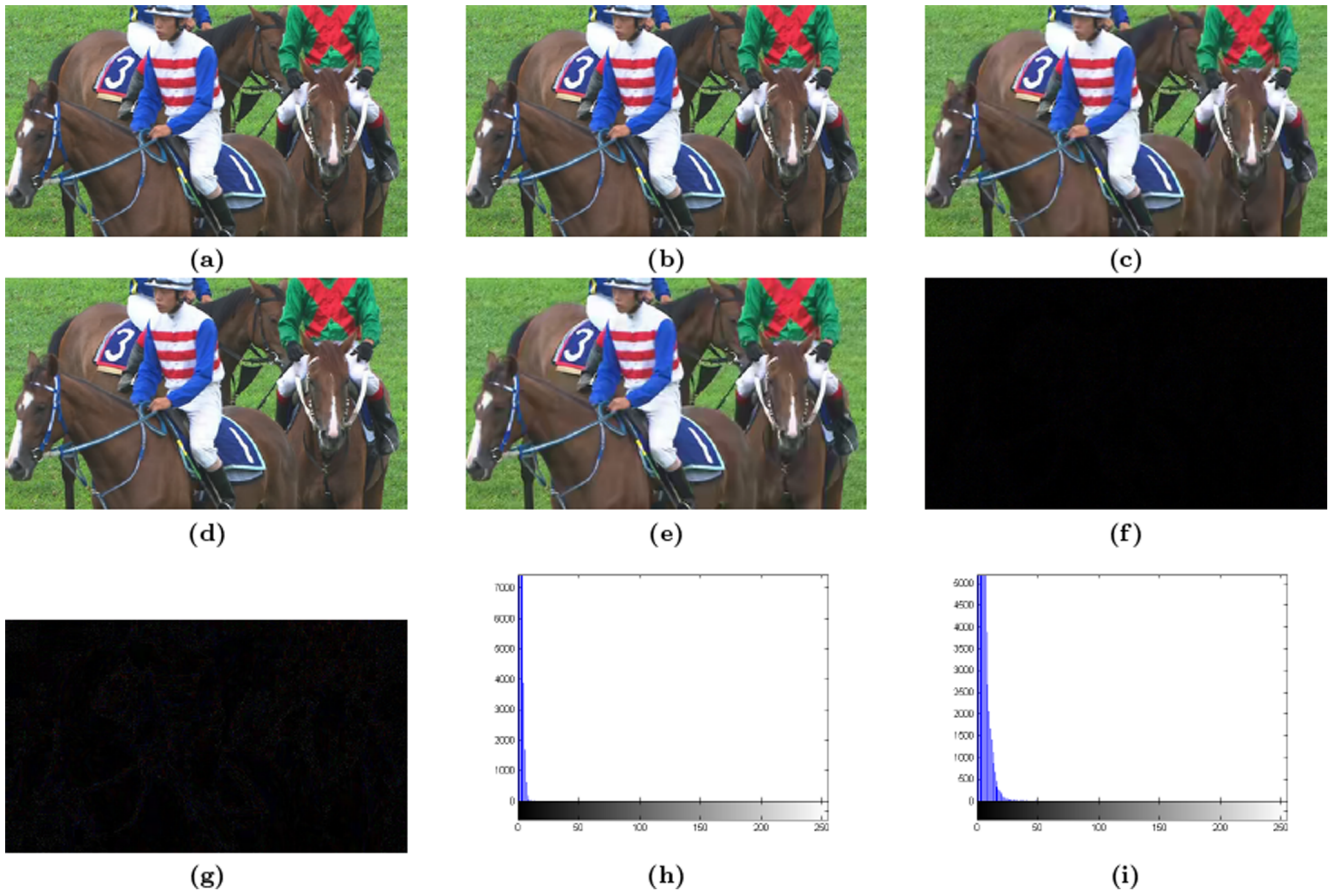
The Racehorses video sequence. (a) Uncompressed video frame, (b) non-watermarked video frames at QP 18, (c) non-watermarked video frames at QP 32, (d) watermarked video frames at QP 18, (e) watermarked video frames at QP 32, (f) the QP 18 difference image (b – d), (g) the QP 32 difference image (c – e), (h) histogram QP 18 difference image (f), and (i) histogram of QP 32 difference image (g).

Table 4 lists the PSNRs of the HEVC coded Y, U and V components at the two QP values with respect to the corresponding original components. PSNRs of the coded components with watermark (WM) and without watermark (Orig.) are given, for the sake of comparison. It can be readily observed that there is not much of the effect on the quality and the maximum we observe is a degradation of 2.27 dB in case of the luma component of RaceHorses at QP 32; the PSNR of 43.42 dB is still not bad par rapport the original 45.69 dB. Figure 3 shows the visual quality of the selected frames of Racehorse video sequence wherein the part (a) shows the uncompressed video, while parts (b) and (c) illustrate the compressed videos at QP 18 and QP 32, respectively. The images in Figure 3.(d) and (e) are the watermarked versions of Figure 3.(b) and (c), respectively. The excellent imperceptibility offered by our method can be gauged by observing parts (f) to (i) of Figure 3. The first two parts are the difference images, which are almost totally black, i.e. the absolute difference between the corresponding pixels are very close to zero; a fact more effectively observed in the histograms given in the last two parts. Note that the histogram par rapport the QP 32 is more drawn out or dilated, because of the fewer coefficients to modify. Still, the escalation is not enough to compromise the visual quality and the PSNR is still high. The imperceptibility aspect is understandable in the face of the fact that the embedding strategy is LSB based. It can be observed from Table 4 that at QP 18 the average decrease in PSNR, over all video sequences, is around 1.03 dB as against 0.28 dB average decrease at QP 32. As far as effect of QP value on the PSNR is concerned, it can be attributed to the fact that, at smaller QP values, the PSNR is generally high due to the presence a greater number of QTCs suitable for watermarking. With higher QP (read 32) values, however, the PSNR decrease is smaller because of the presence of lesser number of coefficients agreeing to the threshold.

**Table 4:** Comparison of PSNR at QP 18 and 32 for watermarked and original video sequences.

QP	Sequences	Y-PNSR		U-PNSR		V-PNSR	
		WM	Orig.	WM	Orig.	WM	Orig.
18	People on Street	45.20	46.28	47.31	47.62	46.93	47.15
	Parkscene	44.02	44.86	45.01	45.68	46.17	46.69
	Chinaspeed	46.26	48.33	47.71	49.38	47.61	49.59
	Vidyo1	46.32	46.81	48.64	48.79	49.41	49.59
	BQmall	43.34	44.87	45.33	45.80	46.51	47.03
	Racehorses	43.42	45.69	44.41	46.06	44.50	46.35
32	People on Street	36.66	36.94	41.37	41.51	41.90	42.02
	Parkscene	35.93	36.06	38.97	39.11	40.28	40.35
	Chinaspeed	36.87	37.87	40.63	41.03	40.05	40.88
	Vidyo1	39.58	39.80	43.89	44.02	44.40	44.57
	BQmall	35.14	35.43	39.20	39.34	40.06	40.15
	Racehorses	34.10	34.39	37.34	37.59	37.64	38.00

When it comes to payload, it will be higher for lower QP values, for obvious reasons. Table 5 confirms that and one can see that at QP 18, the payload is high as we have large number of coefficients agreeing to the threshold and hence a larger number of watermark bits can be embedded. The corresponding payload decreases manifold at QP 32 but there may still be enough number of coefficients in which watermark bits can be embedded.

**Table 5:** Comparison of Payload and Bitrates at QP 18 and 32 for all video sequences.

Sequences	Payload Kbits/Frame		Frame Size (Kbytes)			
	QP 18	QP 32	QP 18		QP 32	
			WM	Orig.	WM	Orig.
People on Street	327.30	38.84	176.03	163.98	37.72	36.60
Parkscene	204.55	15.47	93.58	88.57	15.51	14.89
Chinaspeed	85.32	17.17	30.72	29.29	10.72	10.29
Vidyo1	28.75	3.51	21.45	19.90	4.05	3.91
BQmall	49.81	5.16	49.20	46.91	11.48	11.11
Racehorses	20.07	1.75	7.65	7.12	2.00	1.92

**Figure 4:**
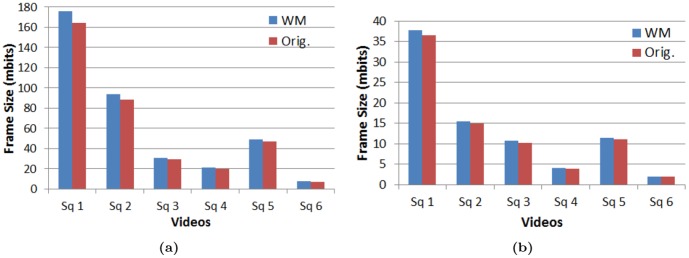
Comparison of Bitrates for watermarked and original video sequences. (a) QP 18 and (b) QP 32.

The frame size escalation is high at lower QP values, as illustrated in Table 5 that shows the frame size comparison at QP 18 and 32 for the video sequences. The average frame size increase is 6.6% for QP 18 as against 3.9% for QP 32. To be more elaborate, Figure 4 illustrates the change in frame size at QP 18 and 32 for varios video sequences. The reason for the increase in bitrate is that the watermarked coefficients are used for reconstruction through the prediction of future block which increase the energy in the residuals thereby escalating the bitrate.

**Figure 5:**
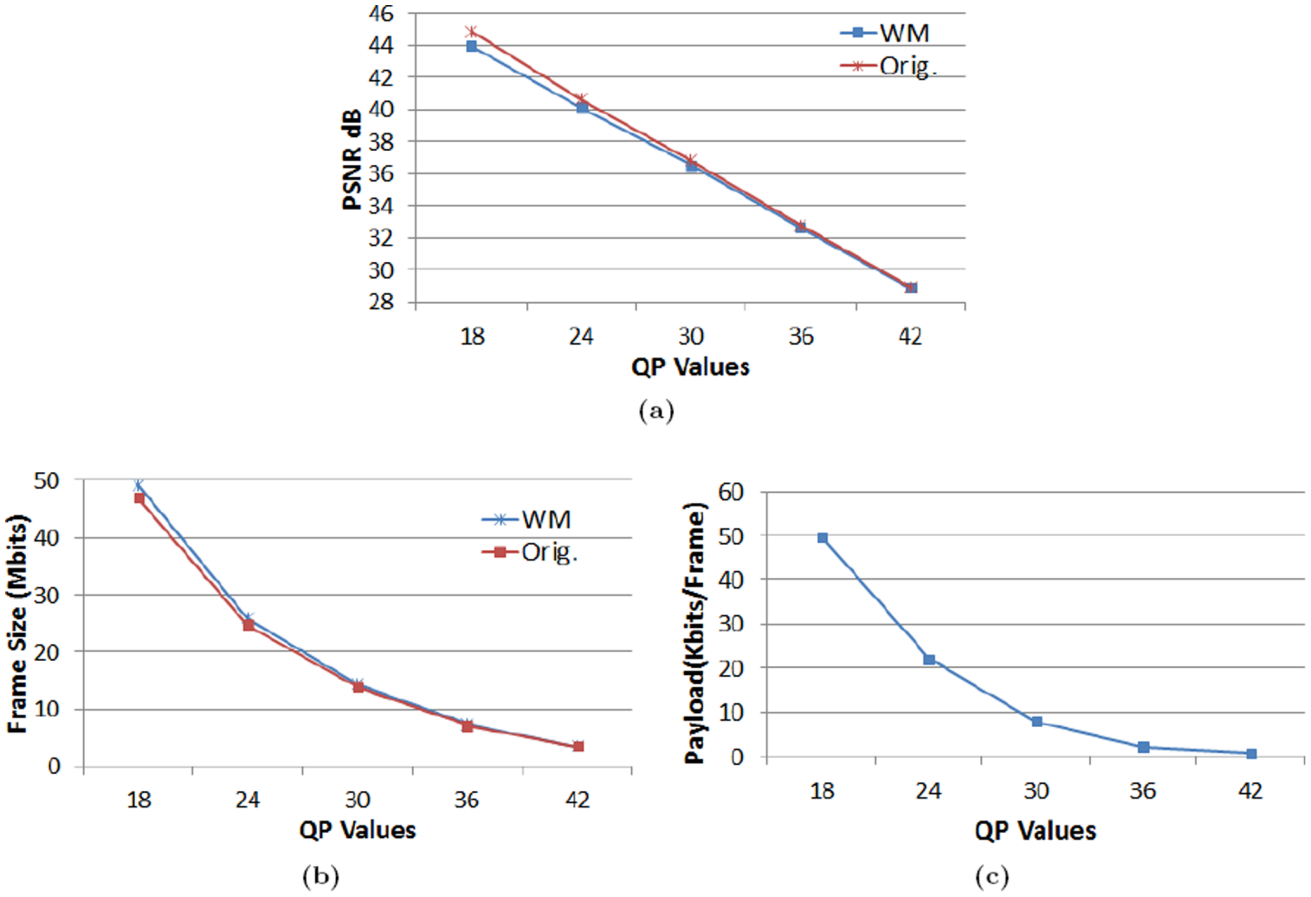
Comparison at whole range of QP for BQMall sequence. (a) PSNR, (b) Frame size and (c) Payload.

Table 6 sums up the overall analyses on PSNR, frame size and Payload in the case of BQMall sequence on the basis of a range of QP values. The ensued trends, illustrated in the form of graphs in Figure 5, establish the following facts:

**Table 6:** Comparison of PSNR, Frame Size and Payload at different range of QP for watermarked and original BQMall video sequence.

QP	PSNR dB		Frame Size Kbytes		Palyload
	WM	Orig.	WM	Orig.	Kbits/frame
18	43.34	44.87	49.20	46.91	49.81
24	40.13	40.66	25.91	24.71	22.09
30	36.36	36.80	14.36	13.81	7.86
36	32.62	32.76	7.35	7.16	2.26
42	28.91	28.96	3.49	3.41	0.71

1. Generally, the PSNR decreases with any increase in the QP value, for both original and watermarked videos. For the video in hand the decreasing function is a straight line. Both the watermarked and un-watermarked video behave the same but, at low QP values, their PSNRs are significantly different from each other; still the watermarked video quality is good.
2. The bitrate escalation is not that significant, as it is already an exponential function of the QP value and the watermark is embedded in the LSBs. The escalation at low QP values is more marked, however.
3. The payload decreases exponentially with respect to an increase in QP value. The lower the QP value, higher will be the payload.

## Conclusion

We proposed a high payload watermarking algorithm for the emerging video coding standard HEVC. For the sake of imperceptibility, the watermark is embedded into the LSBs of selected non-zero coefficients from the QTC domain. The results show that the proposed scheme has the advantages of imperceptibility, bitrate conservation and high payload. These advantages are, however, highly sensitive to the QP value. The escalations are, however, somewhat marked only when the QP value is low. In future, the robustness of the method needs to be improved and a spread spectrum strategy would be explored for embedding.
